# An immune-related seven-gene signature for predicting lymph node metastasis in breast cancer

**DOI:** 10.3389/fmolb.2025.1615524

**Published:** 2025-05-30

**Authors:** Yun Hu, Lanqiao Sun, Jinhua Wang, Yuan Ji, Lili Feng

**Affiliations:** ^1^ Department of Breast Surgery, Jiangsu Cancer Hospital, The Affiliated Cancer Hospital of Nanjing Medical University, Jiangsu Institute of Cancer Research, Nanjing, China; ^2^ Department of General Surgery, Jiangsu Cancer Hospital, The Affiliated Cancer Hospital of Nanjing Medical University, Jiangsu Institute of Cancer Research, Nanjing, China; ^3^ Department of Female Tumor, Jiangsu Cancer Hospital, The Affiliated Cancer Hospital of Nanjing Medical University, Jiangsu Institute of Cancer Research, Nanjing, China; ^4^ Department of Thoracic Surgery, Jiangsu Cancer Hospital, The Affiliated Cancer Hospital of Nanjing Medical University, Jiangsu Institute of Cancer Research, Nanjing, China

**Keywords:** breast cancer, immune, lymph node metastasis, the cancer genome atlas, gene expression omnibus

## Abstract

**Background:**

Breast cancer (BC) is the leading malignant tumors among females worldwide, which serves as a common chronic disease with several acute postoperative complications, including upper limb edema, hemorrhage, flap necrosis, effusion and so on. A majority of BC patients have lymph node metastasis, suffering from a poor prognosis. The immune system has been reported to participate in regulating BC lymph node metastasis. This study aimed to search for immune-related biomarkers for predicting BC lymph node metastasis.

**Methods:**

1057 BC patients were acquired from The Cancer Genome Atlas (TCGA) database as the training dataset while 327 BC patients were obtained from GSE20685 as the validation dataset. We get 2,175 immune genes from four immune-related gene sets. We divided BC patients into lymph node positive and negative groups to identify immune-related lymph node-associated differentially expressed genes (DEGs) for functional enrichment analysis and protein-protein interaction (PPI) network. In order to predict BC lymph node metastasis, we established an immune-related signature and assessed its predictive accuracy. In addition, we applied qRT-PCR to investigate signature gene expressions between normal breast epithelium cells and breast cancer cells.

**Results:**

We identified 336 immune-related lymph node-associated DEGs, which were enriched in leukocyte migration, immunoglobulin complex and receptor ligand activity among GO analysis and cytokine-cytokine receptor interaction among KEGG analysis. With the aim of predicting BC lymph node metastasis, we established a seven-gene immune-related signature, consisting of F2R, IKZF2, NAB1, RFX5, S100B, S1PR2 and VEGFA. The immune-related signature was proven to be an independent predictive factor for BC lymph node metastasis in both TCGA and GSE20685 databases. Compared with normal breast epithelium cells, RFX5, VEGFA were upregulated in breast cancer cells, IKZF2, NAB1, S100B were downregulated in breast cancer cells while F2R, S1PR2 showed no significance.

**Conclusion:**

We established a seven-gene immune-related signature for predicting lymph node metastasis in BC, which might provide a novel sight for BC diagnosis and treatment.

## Introduction

Breast cancer (BC) is the leading malignant tumors among females worldwide, which serves as a common chronic disease with several acute postoperative complications, including upper limb edema, hemorrhage, flap necrosis, effusion and so on (Miller et al., 2021). The lymph nodes serve as an important immune defense line in human body, playing a key role in tumor monitoring and immune activation. BC patients with lymph node metastasis always have larger surgical scopes and poorer survival outcomes. In spite of advanced medical technologies, many BC patients still burden the risk of lymph node metastasis, suffering from a poor prognosis (Liu et al., 2014). Therefore, it is of great importance to identify useful biomarkers for predicting lymph node metastasis in BC. Some researches have been performed to explore biomarkers associated with BC lymph node metastasis. Xu applied contrast-enhanced ultrasound image features to predict BC axillary lymph node metastasis, including peripheral acoustic halo, blood flow classification, ratio of length to diameter and so on, while Mao constructed a radiomics nomogram based on dynamic contrast-enhanced (DCE)-MRI to predict BC axillary lymph node metastasis (Xu et al., 2021; Mao et al., 2020). However, with the aim for early diagnosis of BC lymph node metastasis, more and more further investigations were still of great importance.

Most recently, an increasing number of researches have indicated the important role of immune system for regulating lymph node metastasis in various malignant tumors. It was recorded that lymph node metastasis of papillary thyroid carcinoma (PTC) was related with high CLDN10 expression, which was positively related with immune cells, including B cells, CD8^+^ T cells and macrophages (Xiang et al., 2020). Besides, the increased density of GRANZYME-B+ lymphocytes was discovered in metastatic lymph nodes than corresponding primary tumor in PTC while the level of systemic immune-inflammation index (SII) was an independent predictive factor for central lymph node metastasis in cN0 PTC patients (Cunha et al., 2017; Zhang Z. et al., 2021). According to researches in endometrioid endometrial adenocarcinoma (EEA), several immune system signaling pathways were suppressed during the development of lymph node metastasis, consisting of antigen presentation, cytotoxicity, lymphoid compartment and so on (Cheng et al., 2020). Meanwhile, in head and neck squamous cell carcinoma, lymph node metastasis was associated with antigen changes in primary tumor, following the alternations of antitumor immune response (Kumagai et al., 2010). In addition, Xie applied hyperion imaging system to describe the landscape of immune microenvironment in four oral aquamous cell carcinoma patients, suggesting that twenty-five distinct immune cell subsets were identified and decreased CD8^+^ T cells were found in all patients (Xie et al., 2021). Moreover, the tumor infiltrative growth pattern in stage T1 esophageal squamous cell carcinoma was related with immunosuppression, functioning as an independent factor for lymph node metastasis while lymph node metastasis in primary liver cancer was considered as a risk factor for hyperprogressive disease in patients treated with immune checkpoint inhibitors (Zhao et al., 2020; Xiao et al., 2021). Additionally, the eight-gene immunerelated signature was constructed in lung adenocarcinoma for predicting lymph node metastasis, with risk scores associated with immune cell infiltration, immune scores and immune checkpoint genes (Jia et al., 2021). However, the relationship between immune system and lymph node metastasis in breast cancer remains unclear and deserves future exploration.

With the rapid development of bioinformatics, an increasing number of researchers begin to search for useful biomarkers in public databases, for example, The Cancer Genome Atlas (TCGA) database, Gene Expression Omnibus (GEO) database and so on. In this study, we acquired BC transcriptome and clinical data from the TCGA and GEO databases, with TCGA database as the training dataset and GSE20685 database as the validation dataset. We identified 336 immune-related lymph node-associated differentially expressed genes (DEGs), which were further analyzed by functional enrichment analysis and protein-protein interaction network. We established and verificated a seven-gene immune-related signature for predicting lymph node metastasis in BC, consisting of F2R, IKZF2, NAB1, RFX5, S100B, S1PR2 and VEGFA. Besides, expressions of hub genes between BC patients with different lymph node status were analyzed while correlations between hub genes and immune cells were also performed. In summary, we believed that the immune-related signature for BC lymph node metastasis was of significance, which would provide a novel sight for BC treatment.

## Materials and methods

### Data acquisition and study design

**FIGURE 1:**
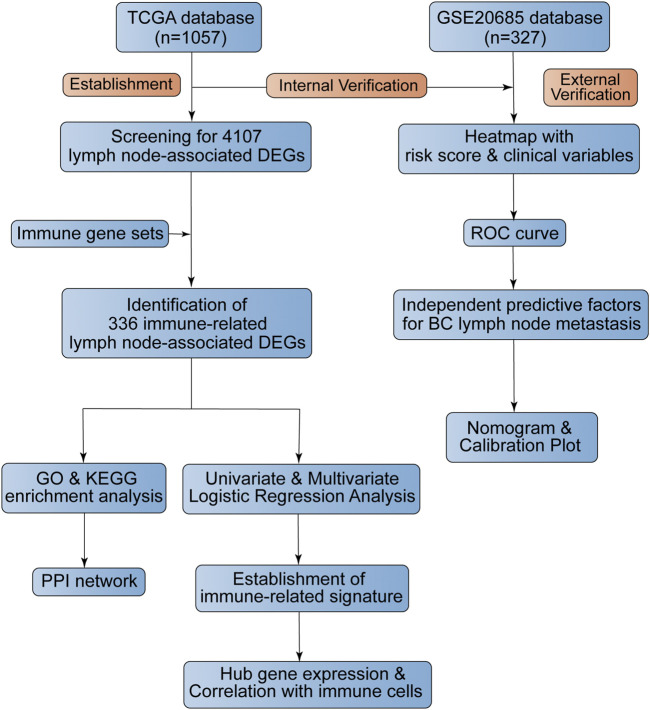
The flowchart of this study.

We downloaded the BC transcriptome and clinical data from the TCGA database (https://portal.gdc.cancer.gov/), which acted as the training dataset. We downloaded an expression profile (GSE20685) from the GEO database (https://www.ncbi.nlm.nih.gov/geo/), which served as the validation dataset. The platform for TCGA transcriptome data was HTSeq-FPKM while the platform for GSE20685 was the GPL570 (HG-U133_Plus_2) Affymetrix Human Genome U133 Plus 2.0 Array. We acquired immune genes from several gene sets, consisting of 29 immune-associated gene sets, “IMMUNE_RESPONSE” (M19817) gene set, “IMMUNE_SYSTEM_PROCESS” (M13664) gene set in Molecular Signatures Database (https://www.gsea-msigdb.org/gsea/msigdb/index.jsp) and “Gene Lists” gene set in ImmPort Database (https://www.immport.org/). The flowchart of our study design was shown in Figure 1.

### Differential expression analysis, functional enrichment analysis and PPI network construction

BC patients were divided into lymph node positive and negative groups and we applied differential expression analysis to identify lymph node-associated differentially expressed genes (DEGs), which was performed by “limma” package and presented through the volcano plot and heatmap. Meanwhile, we acquired immune-related genes by getting union of four gene sets, including 29 immune-associated gene sets, IMMUNE_RESPONSE gene set, IMMUNE_SYSTEM_PROCESS gene set and ImmPort gene set. Furthermore, we screened for immune-related lymph node-associated DEGs by getting the intersection of immune-related genes and lymph node-associated DEGs, which was visualized by the “Venn Diagram” package and “UpSetR” package. In addition, we performed Gene Ontology (GO) and Kyoto Encyclopedia of Genes and Genomes (KEGG) analysis to investigate the potential functions of immune-related lymph node-associated DEGs via “cluster Profiler”, “enrichplot”, “org.Hs.eg.db” and “ggplot2” packages. Besides, we used the STRING database (http://string-db.org) to build a protein-protein interaction (PPI) network with disconnected nodes hided and confidence of 0.950 while top 15 hub genes were selected via “cytoHubba” module in cytoscape software according to the “Degree” score.

### Immune-related signature establishment and verification

We firstly utilized univariate Logistic regression analysis to identify immune-related DEGs associated with BC lymph node metastasis with the criteria of P value <0.001. Then we put identified immune-related DEGs with P value <0.001 into multivariate Logistic regression analysis to eatablish an immune-related signature for predicting BC lymph node metastasis, according to the formula: risk score = Σ(regression coefficient (β) * gene expression). We calculated the risk score of BC patients and divided BC patients into high and low risk groups based on the median value. We presented the expression of genes in immune-related signature between high risk and low risk BC patients by the heatmap while we used Chi-square test to investigate the relationship between risk score and BC clinical features. The predictive value of the immune-related signature for predicting BC lymph node metastasis was analyzed by the receiver operating characteristic (ROC) curve while we applied Logistic regression analysis to screen for independent predictive factors for BC lymph node metastasis. Based on independent predictive factors of BC lymph node metastasis, we built a nomogram via “rms” package and performed the calibration plot to test its predictive value. In addition, we applied GSE20685 as a validation dataset for further verification.

### Expression level of hub genes between patients with different lymph node status

We divided BC patients into lymph node positive and negative groups while we utilized wilcoxon test to investigate the expressions of hub genes in immune-related signature among lymph node positive and negative groups, which were displayed by vioplots through “ggpubr” package. Meanwhile, BC patients were also divided into four subgroups, including N0, N1, N2 and N3 subgroups while we displayed hub gene expressions among four subgroups by “ggpubr” package according to the wilcoxon test.

### Correlation of hub genes with immune cells

We conducted Pearson correlation analysis between seven hub genes and twenty-two immune cells, consisting of B cells naive, B cells memory, Plasma cells, T cells CD8, T cells CD4 naive, T cells CD4 memory resting, T cells CD4 memory activated, T cells follicular helper, T cells regulatory, T cells gamma delta, NK cells resting, NK cells activated, Monocytes, Macrophages M0, Macrophages M1, Macrophages M2, Dendritic cells resting, Dendritic cells activated, Mast cells resting, Mast cells activated, Eosinophils and Neutrophils, which was further visualized by radar plot via “fmsb” package. Moreover, immune cells exhibiting the most remarkable correlation coefficient with seven hub genes were further presented via “ggplot2”, “ggpubr”, “ggExtra” packages.

### Expression of hub genes between normal breast epithelium cells and breast cancer cells

We applied qRT-PCR method to explore hub gene expressions between normal breast epithelium cells and breast cancer cells. Total RNA of normal breast epithelium cells MCF10A and breast cancer cells MDA-MB-231 was extracted through RNA-easy Isolation Reagent (Vazyme) while cDNA was then synthesized through HiScript II Q RT SuperMix (Vazyme) for further qRT-PCR analysis. The process of qRT-PCR included denaturation, hybridization and extension while the temperature change was 95°C to 60°C to 72°C. We applied GAPDH as the normalization control. The primer sequences of hub genes were shown as follows: F2R-forward:CCGCAGGCCAGAATCAAAAG; F2R-reverse: TCATTGGGGTTCCTGAGAAGA; IKZF2-forward: AGCAGCCTAGAAGAACCCCTA; IKZF2-reverse: CAATGCAAACCATGCCACAGA; NAB1-forward: CCACTGACTTCCCTTCCTGTC; NAB1-reverse: GGCAGCACATTTGGGGATTTT; RFX5-forward: TCTCTACCTTCAGCTCCCCTC; RFX5-reverse: ACAGGTGTCAGTGTGCTCTTC; S1PR2-forward: GCCTTCATCGTCATCCTCTGT; S1PR2-reverse: CGAGTGGAACTTGCTGTTTCG; S100B-forward: GGAGACAAGCACAAGCTGAAG; S100B-reverse: CCACAACCTCCTGCTCTTTGA; VEGFA-forward: ATGCGGATCAAACCTCACCAA; VEGFA-reverse: CGCTTTCGTTTTTGCCCCTTT.

### Statistical analysis

We conducted data analysis based on R software and SPSS software. We performed differential expression analysis through “limma” package while we established an immune-related signature for predicting BC lymph node metastasis through univariate and multivariate Logistic regression analysis. We applied comparative analysis by using Wilcoxon test for continuous data and Chi-square test for categorical data. We conducted correlation analysis by using Pearson correlation analysis. The P value <0.05 was statistically significant.

## Results

### Clinical features of breast cancer patients in training and validation datasets

A total of 1057 BC patients in the training dataset was acquired from the TCGA database, with clinical features of age, gender and AJCC-TNM stage. A total of 327 BC patients in the validation dataset was obtained from the GSE20685 database, with clinical features of age, gender and AJCC-TNM stage. All data were available in public and the detailed clinical features of BC patients in training and validation datasets were shown in Table 1.

**TABLE 1:** Clinical features of patients in TCGA and GSE20685 datasets.

Variable	Number, n (%)	
	TCGA database	GSE20685 database
Age	0 patient missing	0 patient missing
<58 years	501 (47.4)	265 (81.0)
≥58 years	556 (52.6)	62 (19.0)
Gender	0 patient missing	0 patient missing
Female	1,045 (98.9)	327 (100)
Male	12 (1.1)	0 (0)
Stage	14 (1.4) patients missing	-
I	177 (16.7)	-
II	606 (57.3)	-
III	243 (23.0)	-
IV	17 (1.6)	-
T	1 (0.1) patient missing	0 patient missing
T1	269 (25.4)	101 (30.9)
T2	614 (58.1)	188 (57.5)
T3	135 (12.8)	26 (8.0)
T4	38 (3.6)	12 (3.6)
N	0 patient missing	0 patient missing
N0	510 (48.2)	137 (41.9)
N1	353 (33.4)	87 (26.6)
N2	120 (11.4)	63 (19.3)
N3	74 (7.0)	40 (12.2)
M	150 (14.2) patient missing	0 patient missing
M0	888 (84.0)	319 (97.6)
M1	19 (1.8)	8 (2.4)

### Identification of immune-related lymph node-associated DEGs

**FIGURE 2:**
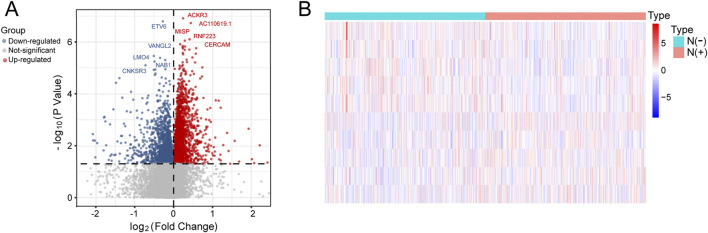
Identification of lymph node-related DEGs. **(A)** The volcano plot of lymph node-related DEGs. **(B)** The heatmap of top 5 upregulated and downregulated lymph node-related DEGs.

**FIGURE 3:**
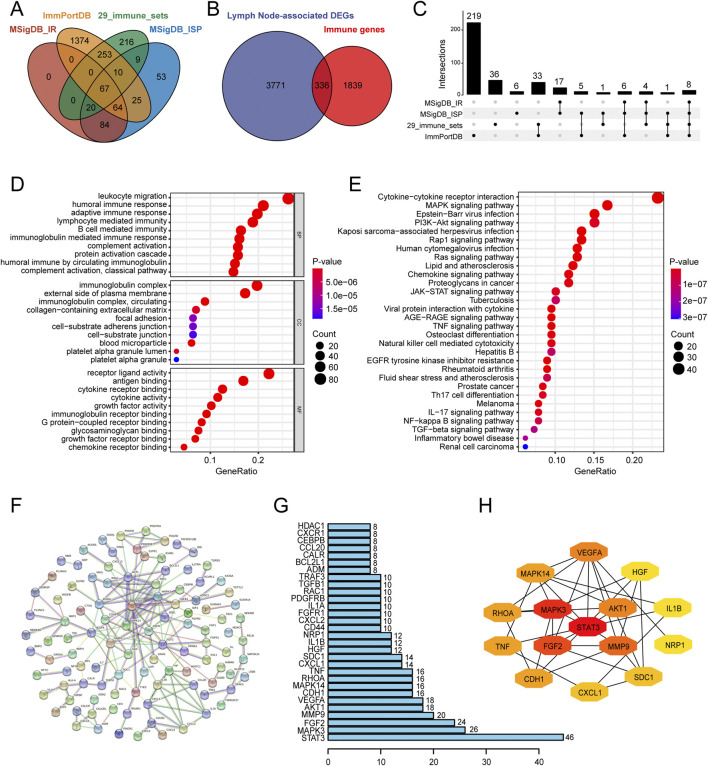
Identification, enrichment analysis and PPI network of immune-related lymph node-associated DEGs. **(A)** The venn diagram showing four immune gene sets. **(B)** The venn diagram identifying 336 immune-related lymph node-associated DEGs. **(C)** The Upset diagram showing the distribution of 336 immune-related lymph node-associated DEGs in four immune gene sets. **(D)** Gene Ontology (GO) enrichment analysis of 336 immune-related lymph node-associated DEGs, including biological process (BP), cellular component (CC) and molecular function (MF) categories. **(E)** Kyoto Encyclopedia of Genes and Genomes (KEGG) enrichment analysis of 336 immune-related lymph node-associated DEGs. **(F)** The PPI network of 336 immune-related lymph node-associated DEGs with disconnected nodes hided. **(G)** Top 30 hub genes in PPI network. **(H)** Top 15 hub genes in PPI network was further visualized by Cytoscape software via “CytoHubba” module. PPI, protein-protein interaction; DEGs, differentially expressed genes.

We performed differential expression analysis to identify 4,107 lymph node-associated DEGs between lymph node positive and negative groups (Figure 2A). The top 5 upregulated and downregulated lymph node-associated DEGs were marked in the volcano plot and further visualized in the heatmap (Figure 2B). Meanwhile, 2,175 immune-related genes were acquired through getting union of four immune gene sets, consisting of 29 immune-associated gene sets, IMMUNE_RESPONSE gene set, IMMUNE_SYSTEM_PROCESS gene set and ImmPort gene set (Figure 3A). Then, we identified 336 immune-related lymph node-associated DEGs by getting the intersection of lymph node associated DEGs and immune genes (Figure 3B). Moreover, we used the Upset diagram to display the distribution of 336 immune-related lymph node-associated DEGs among four immune gene sets (Figure 3C).

### Functional enrichment analysis and PPI network of immune-related lymph node-associated DEGs

In order to investigate potential biological features of 336 immune-related lymph node-associated DEGs, we performed GO and KEGG functional enrichment analysis. According to GO analysis, immune-related lymph node-associated DEGs were mainly enriched in leukocyte migration, immunoglobulin complex and receptor ligand activity among biological process (BP), cellular component (CC) and molecular function (MF) categories respectively (Figure 3D). Besides, KEGG analysis suggested that cytokine-cytokine receptor interaction was mainly enriched (Figure 3E). In addition, we constructed a PPI network of 336 immune-related lymph node-associated DEGs through the STRING database with confidence of 0.950 and disconnected nodes hided, which contained 105 nodes and 338 edges (Figure 3F). Based on the intensity of protein-protein interaction, we displayed top 30 immune-related lymph node-associated genes via bar chart while we presented the sub-network of top 15 immune-related lymph node-associated hub genes according to the “Degree” score (Figures 3G,H).

### Establishment and verification of the immune-related signature

**FIGURE 4:**
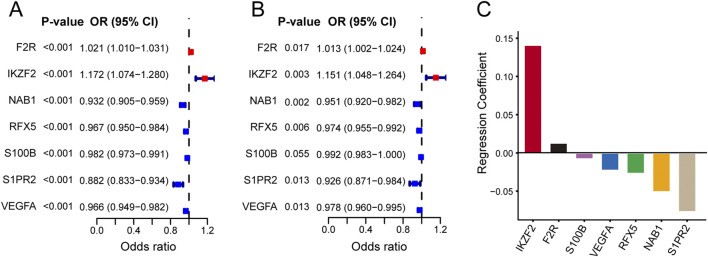
Establishment of the seven-gene immune-related signature. **(A)** The forest plot showing univariate Logistic regression analysis of seven hub genes. **(B)** The forest plot showing multivariate Logistic regression analysis of seven hub genes. **(C)** Regression coefficients of seven hub genes in immune-related signature.

**FIGURE 5:**
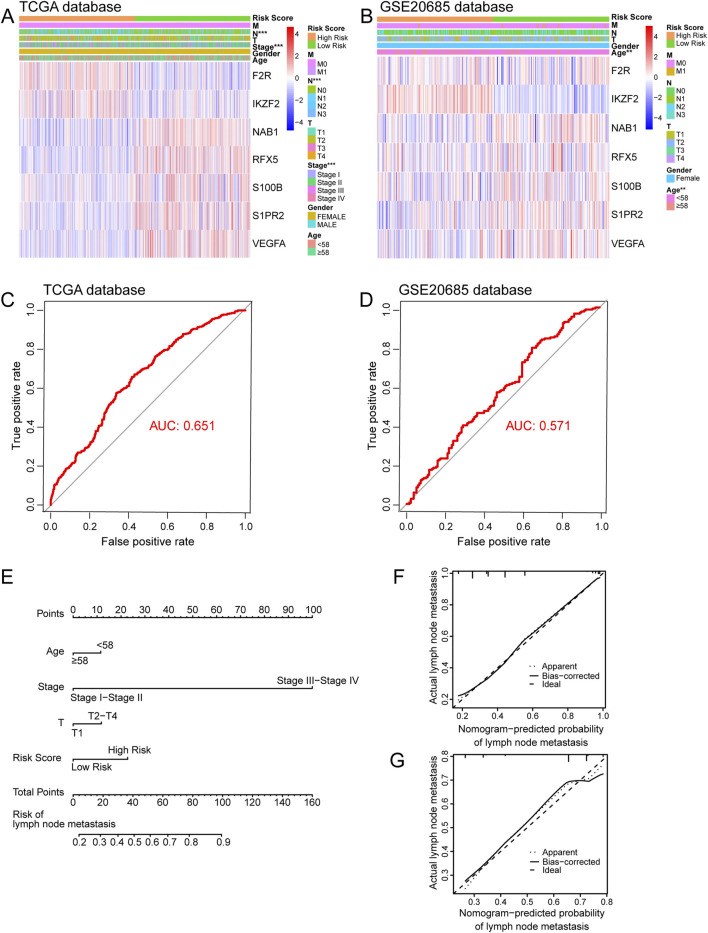
Internal and external verification of immune-related signature. **(A)** The heatmap showing relationship between risk score and clinical variables in TCGA database. **(B)** The heatmap showing relationship between risk score and clinical variables in GSE20685 database. **(C)** The ROC curve of immune-related signature in TCGA database. **(D)** The ROC curve of immune-related signature in GSE20685 database. **(E)** The nomogram for predicting BC lymph node metastasis. **(F)** The calibration plot in TCGA database. **(G)** The calibration plot in GSE20685 database. ROC, receiver operating characteristic.

Firstly, we used univariate Logistic regression analysis to identify 19 genes with P value <0.001 from 336 immune-related lymph node-associated DEGs. Then, we put 19 genes with P value <0.001 into multivariate Logistic regression analysis via the “Forward: LR” method and established a seven-gene immune-related signature for predicting BC lymph node metastasis, including F2R, IKZF2, NAB1, RFX5, S100B, S1PR2 and VEGFA. Based on the regression coefficient and gene expression, we calculated risk scores of BC patients according to the following formula: risk score = exp_F2R_ * (0.013) + exp_IKZF2_ * (0.141) + exp_NAB1_ * (−0.051) + exp_RFX5_ * (−0.027) + exp_S100B_ * (−0.008) + exp_S1PR2_ * (−0.077) + exp_VEGFA_ * (−0.023) (Figure 4) (Table 2). As to the median value of risk scores, BC patients were divided into high and low risk groups. As shown in the heatmap, the expression of NAB1, RFX5, S100B, S1PR2 and VEGFA was higher in low risk group than high risk group while F2R and IKKZF2 exhibited the opposite symptom in both TCGA and GSE20685 databases. Besides, risk score was associated with stage (P < 0.001) and lymph node status (P < 0.001) in TCGA database while risk score displayed correlation with age (P < 0.01) in GSE20685 database (Figures 5A,B). The ROC curve demonstrated the reliable predictive value of the immune-related signature for BC lymph node metastasis in TCGA database (AUC = 0.651) and GSE20685 database (AUC = 0.571) (Figures 5C,D). As to univariate and multivariate Logistic regression analysis, we identified the immune-related signature as an independent predictive factor for BC lymph node metastasis in TCGA database and GSE20685 database (Tables 3,4). Furthermore, we constructed a nomogram for predicting BC lymph node metastasis with all independent predictive factors in TCGA database while we utilized the calibration plot to assess its predictive accuracy in TCGA and GSE20685 databases (Figures 5E-G).

**TABLE 2:** Establishment of a seven-gene immune-related signature.

Gene	Univariate analysis		Multivariate analysis		
	OR (95% CI)	P value	OR (95% CI)	P value	Coefficient
F2R	1.021 (1.010–1.031)	<0.001	1.013 (1.002–1.024)	0.017	0.013
IKZF2	1.172 (1.074–1.280)	<0.001	1.151 (1.048–1.264)	0.003	0.141
NAB1	0.932 (0.905–0.959)	<0.001	0.951 (0.920–0.982)	0.002	−0.051
RFX5	0.967 (0.950–0.984)	<0.001	0.974 (0.955–0.992)	0.006	−0.027
S100B	0.982 (0.973–0.991)	<0.001	0.992 (0.983–1.000)	0.055	−0.008
S1PR2	0.882 (0.833–0.934)	<0.001	0.926 (0.871–0.984)	0.013	−0.077
VEGFA	0.966 (0.949–0.982)	<0.001	0.978 (0.960–0.995)	0.013	−0.023

OR, odds ratio; CI, confidence interval.

**TABLE 3:** Univariate and multivariate Logistic regression analysis for searching independent factors for BC lymph node metastasis in TCGA database.

Variable	Univariate analysis		Multivariate analysis	
	OR (95% CI)	P value	OR (95% CI)	P value
Risk Score	2.825 (2.188–3.648)	<0.001*	2.600 (1.946–3.475)	<0.001*
Age (≥58 years)	0.696 (0.535–0.905)	0.007*	0.619 (0.449–0.854)	0.003*
Gender (Female)	0.377 (0.099–1.429)	0.151	-	-
Stage (Stage III-IV)	46.897 (22.753–96.658)	<0.001*	41.202 (18.915–89.749)	<0.001*
T (T2-T4)	2.435 (1.784–3.322)	<0.001*	1.620 (1.136–2.311)	0.008*
M (M1)	16.293 (2.151–123.386)	0.007*	0.689 (0.076–6.207)	0.740

Age was classified as <58 years and ≥58 years; Gender was classified as female and male; Stage was classified as Stage I-II and Stage III-IV; T was classified as T1 and T2-T4; M was classified as M0 and M1; OR, odds ratio; CI, confidence interval; *, P value <0.05 has statistical significance. BC, breast cancer.

**TABLE 4:** Univariate and multivariate Logistic regression analysis for searching independent factors for BC lymph node metastasis in GSE20685 database.

Variable	Univariate analysis		Multivariate analysis	
	OR (95% CI)	P value	OR (95% CI)	P value
Risk Score	12.432 (1.418–108.979)	0.023*	18.202 (1.780–186.105)	0.014*
Age (≥58 years)	1.394 (0.785–2.475)	0.257	-	-
T (T2-T4)	5.010 (3.019–8.314)	<0.001*	5.197 (3.106–8.698)	<0.001*
M (M1)	-^a^	0.999	-	-

Age was classified as <58 years and ≥58 years; T was classified as T1 and T2-T4; M was classified as M0 and M1; OR, odds ratio; CI, confidence interval; *, P value <0.05 has statistical significance.

^a^ Confidence interval exceeded the limit due to a large P value. BC, breast cancer.

### Differential expression of seven hub genes between patients with different lymph node status

**FIGURE 6:**
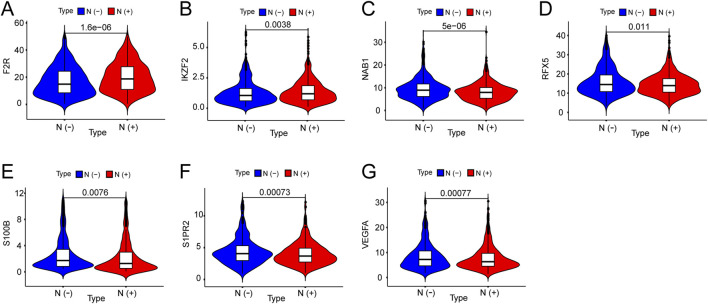
Expression of seven genes among lymph node positive and negative groups in BC. **(A)** F2R expression was positively correlated with BC lymph node metastasis. **(B)** IKZF2 expression was positively correlated with BC lymph node metastasis. **(C)** NAB1 expression was negatively correlated with BC lymph node metastasis. **(D)** RFX5 expression was negatively correlated with BC lymph node metastasis. **(E)** S100B expression was negatively correlated with BC lymph node metastasis. **(F)** SIPR2 expression was negatively correlated with BC lymph node metastasis. **(G)** VEGFA expression was negatively correlated with BC lymph node metastasis.

**FIGURE 7:**
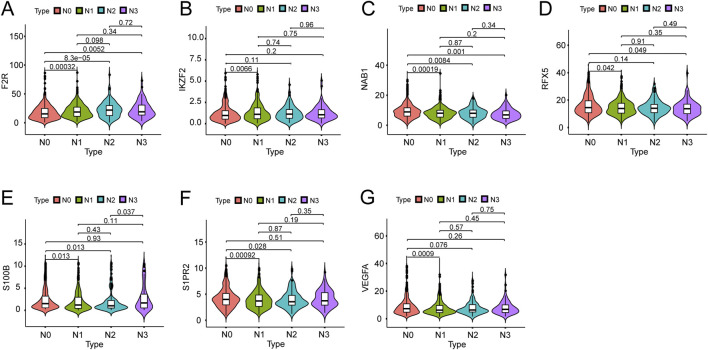
Expression of seven genes among different lymph node groups. **(A)** F2R expression in N0 group was correlated with that in N1, N2 and N3 groups. **(B)** IKZF2 expression in N0 group was correlated with that in N1 group. **(C)** NAB1 expression in N0 group was correlated with that in N1, N2 and N3 groups. **(D)** RFX5 expression in N0 group was correlated with that in N1 and N3 groups. **(E)** S100B expression in N0 group was correlated with that in N1 and N2 groups while S100B expression in N2 group was correlated with that in N3 group. **(F)** S1PR2 expression in N0 group was correlated with that in N1 and N2 groups. **(G)** VEGFA expression in N0 group was correlated with that in N1 group.

We investigated the expression of seven hub genes in the immune-related signature among lymph node positive and negative groups. The expressions of five hub genes were negatively associated with BC lymph node metastasis, including NAB1 (P < 0.001), RFX5 (P = 0.011), S100B (P = 0.008), S1PR2 (P < 0.001) and VEGFA (P < 0.001) while F2R (P < 0.001) and IKZF2 (P = 0.004) showed positive association (Figures 6A–G). Meanwhile, we performed detailed comparative analysis of seven hub genes among N0, N1, N2 and N3 subgroups. F2R expression was lower in N0 group than in N1 (P < 0.001), N2 (P < 0.001) and N3 (P = 0.005) groups while the expression of IKZF2 was lower in N0 group than in N1 group (P = 0.007) (Figures 7A,B). The expression of NAB1 was higher in N0 group than in N1 (P < 0.001), N2 (P = 0.008) and N3 (P = 0.001) groups while RFX5 expression was higher in N0 group than in N1 (P = 0.042) and N3 (P = 0.049) groups (Figures 7C,D). As to S100B, N0 group had higher expression than N1 (P = 0.013) and N2 (P = 0.013) groups while N3 group had higher expression than N2 group (P = 0.037) (Figure 7E). S1PR2 expression was higher in N0 group than in N1 (P < 0.001) and N2 (P = 0.028) groups while the expression of VEGFA was higher in N0 group than in N1 group (P < 0.001) (Figures 7F,G).

### Correlation of seven hubs genes with immune cells

**FIGURE 8:**
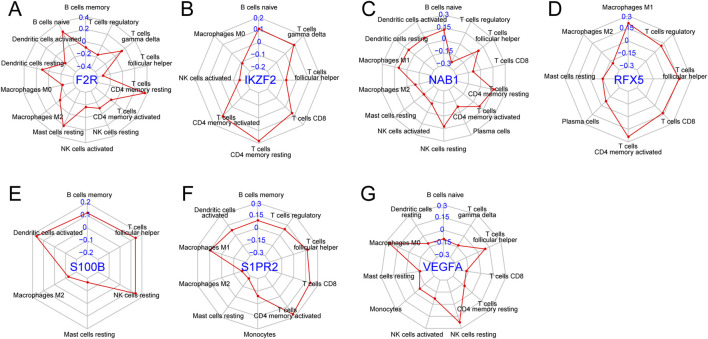
Correlation of seven genes with immune cells. **(A)** Correlation of F2R with fourteen immune cells. **(B)** Correlation of IKZF2 with eight immune cells. **(C)** Correlation of NAB1 with fourteen immune cells. **(D)** Correlation of RFX5 with eight immune cells. **(E)** Correlation of S100B with six immune cells. **(F)** Correlation of S1PR2 with ten immune cells. **(G)** Correlation of VEGFA with eleven immune cells.

**FIGURE 9:**
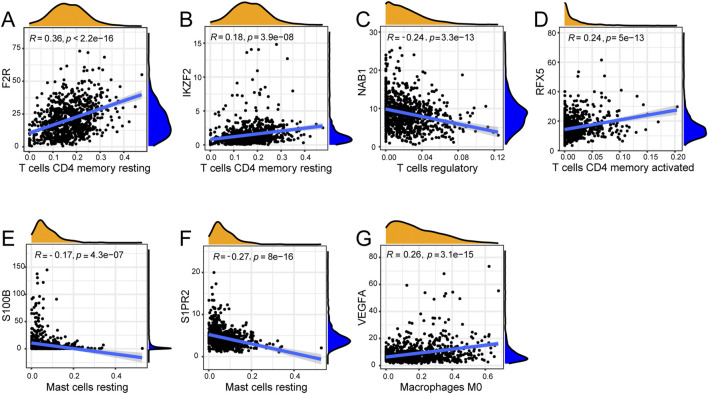
Correlation of seven genes with most remarkable immune cells. **(A)** F2R was most remarkably correlated with T cells CD4 memory resting. **(B)** IKZF2 was most remarkably correlated with T cells CD4 memory resting. **(C)** NAB1 was most remarkably correlated with T cells regulatory. **(D)** RFX5 was most remarkably correlated with T cells CD4 memory activated. **(E)** S100B was most remarkably correlated with Mast cells resting. **(F)** S1PR2 was most remarkably correlated with Mast cells resting. **(G)** VEGFA was most remarkably correlated with Macrophages M0.

We explored the correlation between seven hub genes and twenty-two immune cells. F2R exhibited significant correlations with fourteen immune cells with T cells CD4 memory resting showing the most remarkable correlation coefficient (R = 0.36, P < 0.001) while IKZF2 exhibited significant correlations with eight immune genes with T cells CD4 memory resting presenting the most remarkable correlation coefficient (R = 0.18, P < 0.001) (Figures 8A,B, 9A,B). NAB1 had significant correlations with fourteen immune cells with T cells regulatory showing the most remarkable correlation coefficient (R = −0.24, P < 0.001) while RFX5 had significant correlations with eight immune cells with T cells CD4 memory activated presenting the most remarkable correlation coefficient (R = 0.24, P < 0.001) (Figures 8C,D, 9C,D). S100B displayed six significantly correlated immune cells, S1PR2 displayed ten significantly correlated immune cells while VEGFA showed significant correlations with eleven immune cells (Figures 8E–G). Mast cells resting presented the most remarkable correlation coefficient in S100B (R = −0.17, P < 0.001) and S1PR2 (R = −0.27, P < 0.001)while Macrophages M0 presented the most remarkable correlation coefficient in VEGFA (R = 0.26, P < 0.001) (Figures 9E–G).

### The qRT-PCR verification of seven hub gene expressions between normal breast epithelium cells and breast cancer cells

**FIGURE 10:**
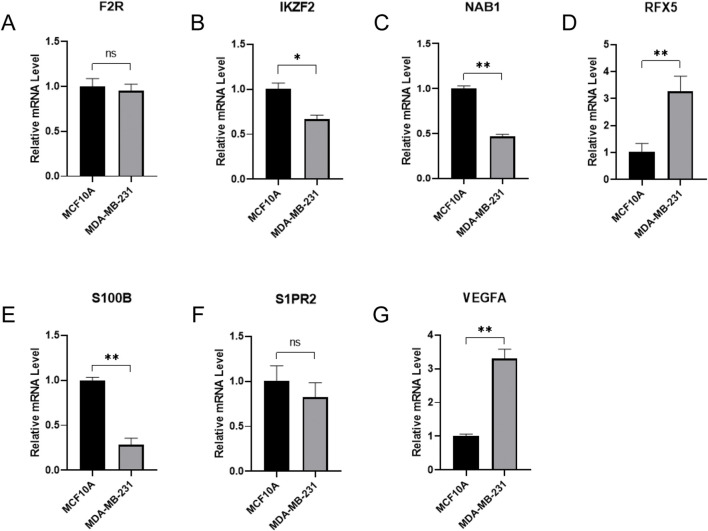
qRT-PCR verification of signature hub gene expressions between normal breast epithelium cells and breast cancer cells. **(A)** F2R expression between normal breast epithelium cells and breast cancer cells. **(B)** IKZF2 expression between normal breast epithelium cells and breast cancer cells. **(C)** NAB1 expression between normal breast epithelium cells and breast cancer cells. **(D)** RFX5 expression between normal breast epithelium cells and breast cancer cells. **(E)** S100B expression between normal breast epithelium cells and breast cancer cells. **(F)** S1PR2 expression between normal breast epithelium cells and breast cancer cells. **(G)** VEGFA expression between normal breast epithelium cells and breast cancer cells.

In order to investigate the expression level of seven hub genes, we applied the qRT-PCR method to analysis their expressions between normal breast epithelium cells and breast cancer cells (Figures 10A–G). Compared with normal breast epithelium cells, the expression levels of RFX5, VEGFA were significantly upregulated in breast cancer cells. Meanwhile, compared with normal breast epithelium cells, the expression levels of IKZF2, NAB1, S100B were significantly downregulated in breast cancer cells. In addition, F2R, S1PR2 showed no significance between normal breast epithelium cells and breast cancer cells.

## Discussion

Breast cancer is one of the leading female malignant tumors in the world, which serves as the second tumor-related death (Siegel et al., 2021). The lymph nodes are recognized as a critical immune defense in human, which were responsible for immune surveillance and immune defense. Despite comprehensive traditional treatments, including surgery, chemotherapy, radiotherapy and so on, a majority of BC patients with lymph node metastasis always suffer from a poor survival outcome (Liu et al., 2015). Therefore, it is very important for clinicians to search for effective biomarkers for predicting lymph node metastasis in BC, which deserves for much attention and futher investigation.

Recently, some researches have investigated the potential relationship between immune system and lymph node metastasis in BC. Tan established an immune-related nomogram for preoperative prediction of axillary lymph node metastasis in triple negative breast cancer (TNBC), whose predictive value was superior to preoperative ultrasound-based axillary lymph node status (Tan et al., 2020). Besides, Zuckerman tried to explore the role of immune cells in tumor draining lymph nodes and discovered the upregulation of tumor-promoting pathways and the downregulation of immune-related pathways in primary tumor, tumor draining lymph nodes and blood of BC patients with positive lymph nodes (Zuckerman et al., 2013). Meanwhile, it is reported that significant increased IDO levels in fresh myeloid-derived suppressor cells were related with BC lymph node metastasis and Foxp3 (+) regulatory T cells infiltration (Yu et al., 2013). On the contrary, during exploring the role of immune infiltrates in peritumoral areas for axillary lymph node metastasis, López suggested that the concentrations of eleven immune markers showed no significant differences between patients with or without axillary lymph node metastasis (López et al., 2020).

In our study, BC data from TCGA database were downloaded as the training dataset while BC data from GSE20685 were acquired as the validation dataset. We obtained immune genes from four immune-related gene sets to further identify 336 immune-related lymph node-associated DEGs, which were enriched in leukocyte migration, immunoglobulin complex, receptor ligand activity and cytokine-cytokine receptor interaction. Furthermore, we established a seven-gene immune-related signature for predicting lymph node metastasis in BC, including F2R, IKZF2, NAB1, RFX5, S100B, S1PR2 and VEGFA. The internal and external verification proved the reliably predictive value of the immune-related signature.

With regard to seven genes in this immune-related signature, some researches have been performed to investigate their potential roles in breast cancer. As to F2R, it was reported that F2R lied in a signaling network, whose inhibition promoted the influence of PI3K pathway inhibitors in TNBC patients lacking of PTEN (Zecchin et al., 2020). According to Diaz, progesterone treatment could enhance the possibility of postmenopausal females for having breast cancer via upregulating F2R expression (Diaz et al., 2012). Besides, Arakaki suggested that F2R-induced Hippo signaling could be inhibited by the tumor suppressor ARRDC3 through sequestration of TAZ in TNBC (Arakaki et al., 2021). As to IKZF2, Tang applied a WGCNA module and MetaDE.ES analysis to obtain thirteen genes for predicting survival risk of BC, including IKZF2 (Tang et al., 2020). In addition, RFX5 was identified in a lncRNA-related coexpression network in BC, which was enriched in glycosphingolipid biosynthesis pathway, transcription regulation and mast cell activation biological processes (Dong et al., 2019). Meanwhile, Hou found that RFX5 could strengthen the transcriptional activity of LINC00504, taking part in BC cell proliferation, migration and invasion (Hou et al., 2021). As to S100B, the suppressive function of S100B for migratory capacity in ER-negative BC was discovered while a high S100B expression was correlated with a good BC overall survival (Yen et al., 2018). However, Bechmann observed BC patients with or without brain metastasis and discovered that serum levels of S100B could not predict the risk for BC brain metastasis (Bechmann et al., 2013). As to VEGFA, the transcription of VEGFA could be suppressed by a small molecule emodin through targeting NCOR2 and SerRS, thus blocking angiogenesis in TNBC-bearing mice (Zou et al., 2020). The analysis of gene polymorphisms indicated that BC patients with VEGFA 2578 C>A had a better overall survival while VEGFA expression in BC was reported to show a significant association with PD-L1 positivity (Madrid-Paredes et al., 2020; Fujii et al., 2020). Besides, Pu demonstrated that VEGFA served as the hub gene in BC treatments of Fluvastatin and Zoledronate through regulating BC migration, invasion and apoptosis (Pu et al., 2018). However, the researches of NAB1 and S1PR2 in BC remain rare and deserve for further investigation. In gastric cancer, NAB1 was positively co-expressed with ZNF860, which functioned as an independent prognostic factor for recurrence-free survival while the internalization of S1PR2 in colorectal cancer could lead to 5-FU resistance by upregulating uracil generation (Pan et al., 2019; Zhang YH. et al., 2021).

However, there are still some limitations in this study. On the one hand, we applied TCGA database and GEO database to establish immune-related signature, clinical data of chinese BC patients should also be enrolled in the future for further verification. On the other hand, we built the immune-related signature through seven hub genes, more experiments *in vivo* or *in vitro* should be applied for investigating potential signaling pathways. In summary, the immune system plays an important role in regulating lymph node metastasis in BC, which is worthy of further investigation. After a comprehensive analysis, we identified a seven-gene immune-related signature for predicting BC lymph node metastasis, which might provide guidance for early diagnosis and effective therapies in BC.

## Conclusion

A seven-gene immune-related signature was established and verificated for predicting lymph node metastasis in BC, consisting of F2R, IKZF2, NAB1, RFX5, S100B, S1PR2 and VEGFA, which might provide guidance for BC diagnosis and treatment.

## Data Availability

The original contributions presented in the study are included in the article/Supplementary Material, further inquiries can be directed to the corresponding authors.

## Abbreviations

BP, biological process; BC, breast cancer; CC, cellular component; DEGs, differentially expressed genes; DCE-MRI, dynamic contrast-enhanced-MRI; EEA, endometrioid endometrial adenocarcinoma; GEO, gene expression omnibus; GO, gene ontology; KEGG, kyoto encyclopedia of genes and genomes; MF, molecular function; PTC, papillary thyroid carcinoma; PPI, protein-protein interaction; ROC, receiver operating characteristic; SII, systemic immune-inflammation index; TNBC, triple negative breast cancer; TCGA, the cancer genome atlas.

## References

[B1] Arakaki A. K. S. Pan W. A. Wedegaertner H. Roca-Mercado I. Chinn L. Gujral T. S. (2021). α-Arrestin ARRDC3 tumor suppressor function is linked to GPCR-induced TAZ activation and breast cancer metastasis. J. Cell Sci. 134, jcs254888. 10.1242/jcs.254888 33722977 PMC8084569

[B2] Bechmann T. Madsen J. S. Brandslund I. Lund E. D. Ormstrup T. Jakobsen E. H. (2013). Predicting brain metastases of breast cancer based on serum S100B and serum HER2. Oncol. Lett. 6, 1265–1270. 10.3892/ol.2013.1536 24179506 PMC3813762

[B3] Cheng Y. Zhang X. Wang Z. Wang J. (2020). Reconstruction of immune microenvironment and signaling pathways in endometrioid endometrial adenocarcinoma during formation of lymphovascular space involvement and lymph node metastasis. Front. Oncol. 10, 595082. 10.3389/fonc.2020.595082 33363026 PMC7756104

[B4] Cunha L. L. Nonogaki S. Soares F. A. Vassallo J. Ward L. S. (2017). Immune escape mechanism is impaired in the microenvironment of thyroid lymph node metastasis. Endocr. Pathol. 28, 369–372. 10.1007/s12022-017-9495-2 28730569

[B5] Diaz J. Aranda E. Henriquez S. Quezada M. Espinoza E. Bravo M. L. (2012). Progesterone promotes focal adhesion formation and migration in breast cancer cells through induction of protease-activated receptor-1. J. Endocrinol. 214, 165–175. 10.1530/JOE-11-0310 22593082

[B6] Dong Y. Zhang T. Li X. Yu F. Guo Y. (2019). Comprehensive analysis of coexpressed long noncoding RNAs and genes in breast cancer. J. Obstet. Gynaecol. Res. 45, 428–437. 10.1111/jog.13840 30362198

[B7] Fujii T. Hirakata T. Kurozumi S. Tokuda S. Nakazawa Y. Obayashi S. (2020). VEGF-A is associated with the degree of TILs and PD-L1 expression in primary breast cancer. Vivo 34, 2641–2646. 10.21873/invivo.12082 PMC7652471 32871794

[B8] Hou T. Ye L. Wu S. (2021). Knockdown of LINC00504 inhibits the proliferation and invasion of breast cancer via the downregulation of miR-140-5p. Onco. Targets. Ther. 14, 3991–4003. 10.2147/OTT.S294965 34239305 PMC8259944

[B10] Jia R. Sui Z. Zhang H. Yu Z. (2021). Identification and validation of immune-related gene signature for predicting lymph node metastasis and prognosis in lung adenocarcinoma. Front. Mol. Biosci. 8, 679031. 10.3389/fmolb.2021.679031 34109216 PMC8182055

[B11] Kumagai K. Hamada Y. Gotoh A. Kobayashi H. Kawaguchi K. Horie A. (2010). Evidence for the changes of antitumor immune response during lymph node metastasis in head and neck squamous cell carcinoma. Oral Surg. Oral Med. Oral Pathol. Oral Radiol. Endod. 110, 341–350. 10.1016/j.tripleo.2010.03.030 20598595

[B12] Liu D. Chen Y. Deng M. Xie G. Wang J. Zhang L. (2014). Lymph node ratio and breast cancer prognosis: a meta-analysis. Breast Cancer 21, 1–9. 10.1007/s12282-013-0497-8 24101545

[B13] Liu X. H. Zhang L. Chen B. (2015). A meta-analysis of the prognosis in patients with breast cancer with ipsilateral supraclavicular lymph node metastasis versus patients with stage IIIb/c or IV breast cancer. Chronic Dis. Transl. Med. 1, 236–242. 10.1016/j.cdtm.2016.01.002 29063013 PMC5643591

[B14] López C. Bosch-Príncep R. Orero G. Fontoura Balagueró L. Korzynska A. García-Rojo M. (2020). Peritumoral immune infiltrates in primary tumours are not associated with the presence of axillary lymph node metastasis in breast cancer: a retrospective cohort study. PeerJ 8, e9779. 10.7717/peerj.9779 32953267 PMC7474517

[B15] Madrid-Paredes A. Casado-Combreras M. Pérez-Ramírez C. Segura-Pérez A. M. Chamorro-Santos C. Vergara-Alcalde E. (2020). Association of ABCB1 and VEGFA gene polymorphisms with breast cancer susceptibility and prognosis. Pathol. Res. Pract. 216, 152860. 10.1016/j.prp.2020.152860 32127237

[B16] Mao N. Dai Y. Lin F. Ma H. Duan S. Xie H. (2020). Radiomics nomogram of DCE-MRI for the prediction of axillary lymph node metastasis in breast cancer. Front. Oncol. 10, 541849. 10.3389/fonc.2020.541849 33381444 PMC7769044

[B17] Miller K. D. Ortiz A. P. Pinheiro P. S. Bandi P. Minihan A. Fuchs H. E. (2021). Cancer statistics for the US Hispanic/Latino population. Ca. Cancer J. Clin. 71, 466–487. 10.3322/caac.21695 34545941

[B18] Pan H. X. Bai H. S. Guo Y. Cheng Z. Y. (2019). Bioinformatic analysis of the prognostic value of ZNF860 in recurrence-free survival and its potential regulative network in gastric cancer. Eur. Rev. Med. Pharmacol. Sci. 23, 162–170. 10.26355/eurrev_201901_16760 30767193

[B19] Pu H. Zhang Q. Zhao C. Shi L. Wang Y. Wang J. (2018). VEGFA involves in the use of Fluvastatin and zoledronate against breast cancer. Pathol. Oncol. Res. 24, 557–565. 10.1007/s12253-017-0277-4 28744693

[B20] Siegel R. L. Miller K. D. Fuchs H. E. Jemal A. (2021). Cancer statistics, 2021. Ca. Cancer J. Clin. 71, 7–33. 10.3322/caac.21654 33433946

[B21] Tan W. Xie X. Huang Z. Chen L. Tang W. Zhu R. (2020). Construction of an immune-related genes nomogram for the preoperative prediction of axillary lymph node metastasis in triple-negative breast cancer. Artif. Cells Nanomed Biotechnol. 48, 288–297. 10.1080/21691401.2019.1703731 31858816

[B22] Tang W. Guo X. Niu L. Song D. Han B. Zhang H. (2020). Identification of key molecular targets that correlate with breast cancer through bioinformatic methods. J. Gene Med. 22, e3141. 10.1002/jgm.3141 31697007

[B23] Xiang Z. Zhong C. Chang A. Ling J. Zhao H. Zhou W. (2020). Immune-related key gene CLDN10 correlates with lymph node metastasis but predicts favorable prognosis in papillary thyroid carcinoma. Aging (Albany NY) 12, 2825–2839. 10.18632/aging.102780 32045884 PMC7041783

[B24] Xiao L. S. Li Q. M. Hu C. Y. Cui H. Hong C. Huang C. Y. (2021). Lung metastasis and lymph node metastasis are risk factors for hyperprogressive disease in primary liver cancer patients treated with immune checkpoint inhibitors. Ann. Palliat. Med. 10, 11244–11254. 10.21037/apm-21-2023 34670386

[B25] Xie S. Zhang X. Y. Shan X. F. Yau V. Zhang J. Y. Wang W. (2021). Hyperion image analysis depicts a preliminary landscape of tumor immune microenvironment in OSCC with lymph node metastasis. J. Immunol. Res. 2021, 9975423. 10.1155/2021/9975423 34239944 PMC8238606

[B26] Xu H. Xu G. L. Li X. D. Su Q. H. Dong C. Z. (2021). Correlation between the contrast-enhanced ultrasound image features and axillary lymph node metastasis of primary breast cancer and its diagnostic value. Clin. Transl. Oncol. 23, 155–163. 10.1007/s12094-020-02407-6 32488804

[B27] Yen M. C. Huang Y. C. Kan J. Y. Kuo P. L. Hou M. F. Hsu Y. L. (2018). S100B expression in breast cancer as a predictive marker for cancer metastasis. Int. J. Oncol. 52, 433–440. 10.3892/ijo.2017.4226 29345293

[B28] Yu J. Du W. Yan F. Wang Y. Li H. Cao S. (2013). Myeloid-derived suppressor cells suppress antitumor immune responses through IDO expression and correlate with lymph node metastasis in patients with breast cancer. J. Immunol. 190, 3783–3797. 10.4049/jimmunol.1201449 23440412

[B29] Zecchin D. Moore C. Michailidis F. Horswell S. Rana S. Howell M. (2020). Combined targeting of G protein-coupled receptor and EGF receptor signaling overcomes resistance to PI3K pathway inhibitors in PTEN-null triple negative breast cancer. Breast Cancer 12, e11987. 10.15252/emmm.202011987 PMC7411640 32672423

[B30] Zhang Y. H. Cui S. X. Wan S. B. Wu S. H. Qu X. J. (2021b). Increased S1P induces S1PR2 internalization to blunt the sensitivity of colorectal cancer to 5-fluorouracil via promoting intracellular uracil generation. Acta Pharmacol. Sin. 42, 460–469. 10.1038/s41401-020-0460-0 32647340 PMC8027438

[B31] Zhang Z. Xia F. Wang W. Huang Y. Li X. (2021a). The systemic immune-inflammation index-based model is an effective biomarker on predicting central lymph node metastasis in clinically nodal-negative papillary thyroid carcinoma. Gland. Surg. 10, 1368–1373. 10.21037/gs-20-666 33968688 PMC8102221

[B32] Zhao Y. Xu E. Yang X. Zhang Y. Chen H. Wang Y. (2020). Tumor infiltrative growth pattern correlates with the immune microenvironment and is an independent factor for lymph node metastasis and prognosis in stage T1 esophageal squamous cell carcinoma. Virchows Arch. 477, 401–408. 10.1007/s00428-020-02801-z 32232560

[B33] Zou G. Zhang X. Wang L. Li X. Xie T. Zhao J. (2020). Herb-sourced emodin inhibits angiogenesis of breast cancer by targeting VEGFA transcription. Theranostics 10, 6839–6853. 10.7150/thno.43622 32550907 PMC7295066

[B34] Zuckerman N. S. Yu H. Simons D. L. Bhattacharya N. Carcamo-Cavazos V. Yan N. (2013). Altered local and systemic immune profiles underlie lymph node metastasis in breast cancer patients. Int. J. Cancer 132, 2537–2547. 10.1002/ijc.27933 23136075 PMC3609917

